# Comparative genomics and phylogenetic analysis of seven *Ficus* species based on chloroplast genomes

**DOI:** 10.7717/peerj.20531

**Published:** 2026-01-07

**Authors:** SuQing Bao, Lili Deng, YanCai Shi, Na Duan

**Affiliations:** 1 Guilin Medical University, Guilin, Guangxi, China; 2 Guangxi Zhuang Autonomous Region and Chinese Academy of Sciences, Guangxi Institute of Botany, Guilin, Guangxi, China; 3 Changzhi University, Department of Life Sciences, Changzhi, Shanxi, China

**Keywords:** *Ficus*, Comparative genomics, Molecular markers

## Abstract

**Background:**

The genus *Ficus* (Moraceae) is a large and ecologically important group, known for its intricate fig-wasp pollination mutualism and role as a keystone resource in tropical ecosystems. Despite its significance, the phylogenetic relationships within *Ficus* remain partially unresolved, necessitating more comprehensive genomic data. Chloroplast (cp) genomes are valuable resources for plant phylogenetic and comparative genomic studies. Here, we sequenced, assembled, and comparatively analyzed the complete chloroplast genomes of seven *Ficus* species, including *Ficus esquiroliana*, *Ficus pandurata*, *Ficus formosana*, *Ficus erecta*, *Ficus carica*, *Ficus hirta*, and *Ficus stenophylla*.

**Results:**

The complete cp genomes were successfully assembled, ranging in size from 160,340 bp to 160,669 bp, and exhibited a typical quadripartite structure with highly conserved gene content and arrangement. Critically, while some of these species have previously published plastomes, our assemblies consistently encoded 130 genes, contrasting with reported gene counts (*e.g.*, 129 for *F. formosana* (NC_059898), 119 for *F. carica* (KY635880), 131 for *F. erecta* (MT093220)) in earlier studies. Numerous repeat sequences and simple sequence repeats (SSRs) were identified, predominantly in non-coding regions, which serve as valuable resources for developing novel genetic markers. Analysis of codon usage revealed a strong bias towards A/T endings, a common feature in plant cp genomes. While inverted repeat (IR) boundary regions were largely conserved, minor variations, including partial gene duplications (*rps*19, *rpl*2), were observed. Comparative genome alignment and nucleotide diversity analysis showed high sequence conservation, with most variations concentrated in single-copy and non-coding regions. We identified three hypervariable regions (*ccs*A, *ccs*A - *ndh*D, and *rpo*B - *trn*C-GCA) with elevated nucleotide diversity (Pi > 0.012, *ccs*A up to 0.0141), suggesting their utility as candidate DNA barcodes for *Ficus*. Phylogenetic analysis using 79 protein-coding genes from 26 species robustly supported the monophyly of *Ficus* and resolved the seven newly sequenced species into two well-supported clades, consistent with previous classifications.

**Conclusions:**

Our study provides new, consistently assembled and rigorously annotated chloroplast genome data for *Ficus*, including clarified data for previously studied species with notable gene content discrepancies. These data identify candidate molecular markers with potential applications for systematics and population genetics, and offer robust insights into relationships among sampled taxa. These data will facilitate future studies of *Ficus* evolution and conservation when complemented by broader taxon sampling and nuclear/mitochondrial data.

## Background

The genus *Ficus* L. (Moraceae), commonly known as fig trees, comprises one of the largest and most ecologically consequential genera of flowering plants, with over 800 described species largely distributed across tropical and subtropical regions (Berg, 1989; Cruaud et al., 2012). *Ficus* species are notable for the obligate, species-specific pollination mutualism with agaonid fig wasps—a classic model of coevolution and for their prominent role as keystone resources that sustain frugivores and structure tropical and subtropical forest communities (Zhang et al., 2020; Raji & Downs, 2021; Devi et al., 2022). Several species (*e.g.*, *F. carica*) are also of economic and ethnobotanical importance as food and in traditional medicine. Despite broad agreement on the monophyly of *Ficus*, relationships among many infrageneric lineages remain incompletely resolved; causes include ancient rapid radiations, morphological plasticity, hybridization and incomplete lineage sorting (Rønsted et al., 2008; Cruaud et al., 2012; Zhang et al., 2020).

Chloroplast genomes have become a routine and powerful resource for plant phylogenetics and comparative genomics because of their generally conserved gene content and organization, uniparental (often maternal) inheritance, and the presence of variable intergenic and coding regions useful for resolving relationships at a range of taxonomic depths (Jansen et al., 2005; Daniell et al., 2016). Advances in high-throughput sequencing have led to a rapid increase in the number of complete plastomes available across angiosperms and within Moraceae, improving our ability to detect mutational hotspots, design lineage-specific markers, and leverage phylogenomic datasets for resolving difficult nodes (Duan et al., 2022; Zeng et al., 2022).

Over the past few years, multiple studies have applied complete chloroplast genomes to address systematics and marker development within *Ficus*. For example, Huang et al. (2022) performed a comparative plastome analysis of ten *Ficus* species and highlighted mutational hotspot regions and candidate loci for barcoding; Xia et al. (2022) analyzed eight *Ficus* plastomes and provided phylogenomic insights that supported particular subgeneric groupings; Zhang et al., (2022a) and Zhang et al. (2022b) focused on the *F. sarmentosa* species complex and used plastomes to clarify relationships among closely related taxa; Vu et al. (2023) sequenced the plastome of *F. simplicissima* and proposed plastome-derived barcodes for species identification. These investigations consistently show that plastome-scale data increase resolution relative to single-locus markers, but they also reveal that hotspot regions and phylogenetic placements can be sensitive to taxon sampling and geographic representation. In addition, complementary organellar resources are beginning to appear: a recent report of the complete mitochondrial genome of *F. hirta* (Deng & Cai, 2025) underscores the value of integrating multiple organellar genomes for comparative analyses and for detecting potential shared transfers or assembly artifacts. Comparative plastome studies in other Moraceae genera (Zeng et al., 2022) further illustrate the utility of organellar genomes for resolving genus-level taxonomy and for identifying evolutionarily informative markers.

Despite these advances, taxon sampling across the genus remains incomplete: many sections and geographic lineages of *Ficus* are still under-represented in plastome datasets, and variation in sampling strategies complicates straightforward comparisons of hotspot loci among studies. To address this gap and to expand available organellar genomic resources for *Ficus*, we sequenced, assembled and analyzed the complete chloroplast genomes of seven *Ficus* species (*F. esquiroliana*, *F. pandurata*, *F. formosana*, *F. erecta*, *F. carica*, *F. hirta*, and *F. stenophylla*). We conducted comprehensive analyses of genome structure, repeat content, codon usage, sequence divergence, and reconstructed their phylogenetic relationships using plastome data. Our findings provide insights into genome evolution and the phylogenetic framework of *Ficus*, which can facilitate further research on its taxonomy, evolution, and conservation.

## Methods

### Plant material, DNA extraction, and sequencing

All seven *Ficus* species examined in this study (*F. esquiroliana*, *F. pandurata*, *F. formosana*, *F. erecta*, *F. carica*, *F. hirta*, and *F. stenophylla*) were sampled. Voucher specimen information and herbarium accession numbers listed in Table S1; all vouchers are deposited in the Herbarium of Guangxi Institute of Botany. Sampling and collection complied with the regulations of the Guangxi Zhuang Autonomous Region, and consisted of common, non-protected species collected from non-restricted locations.

Total genomic DNA was extracted from approximately 50 mg of silica-dried young leaf tissue using the modified cetyltrimethylammonium bromide (CTAB) method (Doyle & Doyle, 1987). DNA quality and concentration were assessed using agarose gel electrophoresis and a NanoDrop spectrophotometer. High-quality genomic DNA library construction and sequencing were performed by Beijing Gezhi Boya Biotechnology Co., Ltd. DNA was evaluated by Qubit, NanoDrop, and gel electrophoresis, then sonicated using a Covaris S220 to an average insert size of approximately 350 bp. Library construction was performed using the NEBNext^®^ Ultra™ II DNA Library Prep Kit. Sequencing was performed on an Illumina HiSeq system (Illumina, San Diego, CA) using a paired-end 2 × 150 bp configuration.

### Chloroplast genome assembly and annotation

Raw sequencing reads were filtered to remove low-quality reads and adapters using Trimmomatic version 0.36 (Bolger, Lohse & Usadel, 2014). Clean reads were assembled into complete chloroplast genomes using NOVOPlasty v4.3 (Dierckxsens, Mardulyn & Smits, 2017), with the *mat*K gene used as the seed sequence and the complete chloroplast genome of a related *Ficus* species (GenBank accession MN364706) supplied as a reference. The assembly accuracy was confirmed by mapping reads back to the assembled genomes. Annotation was performed with Plann version 1.1 (Huang & Cronk, 2015) and manually corrected in Geneious version 10.2.6 (Kearse et al., 2012). The circular genome map was visualized using OGDRAW version 1.3.1 (http://ogdraw.mpimp-golm.mpg.de/) (Greiner, Lehwark & Bock, 2019).

### Repeat sequence analysis

Simple sequence repeats (SSRs) were detected using MIcroSAtellite version 2.1 (MISA, https://webblast.ipk-gatersleben.de/misa) (Beier et al., 2017) with default parameters (minimum repeat numbers: 10 for mononucleotide, 5 for dinucleotide, 4 for trinucleotide, and 3 for tetra-, penta-, and hexanucleotide repeats). Long repeat sequences (forward, reverse, palindrome, and complementary) were identified using REPuter version 1.1 (https://bibiserv.techfak.uni-bielefeld.de/reputer) (Kurtz et al., 2001) with a minimum repeat size of 30 bp and sequence identity greater than 90%. Tandem Repeats Finder version 4.04 (http://tandem.bu.edu/trf/trf.html) (Benson, 1999) was used for tandem repeat analysis, with parameters set to 2 for the alignment parameter match and 7 for mismatches and indels.

### Codon usage analysis

Protein-coding genes were extracted, and codon usage patterns were analyzed using CodonW version1.4.4 (http://codonw.sourceforge.net/) (Sharp & Li, 1986) to compute the relative synonymous codon usage (RSCU) values. Heatmaps are drawn using TBtools version 2.2.2 (Chen et al., 2020), with RSCU = 1 defined as the neutral (no-bias) value and used as the central point of the color scale (white).

### Comparative genome and sequence divergence analysis

Genomic structure visualization and global alignment among the seven *Ficus* cp genomes were performed using mVISTA (Frazer et al., 2004) in Shuffle-LAGAN mode, with *F. hirta* as the reference. The borders of the large single-copy (LSC), small single-copy (SSC), and inverted repeat (IR) regions were compared and visualized using IRscope version 3.1 (https://links.jianshu.com/go?to=https://irscope.shinyapps.io/irapp/) (Amiryousefi, Hyvönen & Poczai, 2018).

Nucleotide diversity (Pi) was calculated using DnaSP version 6.0 (Rozas et al., 2017) with a sliding window analysis (window size: 600 bp; step size: 200 bp) to identify highly variable regions among the cp genomes.

### Phylogenetic analysis

Phylogenetic analyses were conducted on a concatenated nucleotide alignment of 79 complete chloroplast protein-coding genes (PCGs) from 26 Moraceae taxa (Table S2); *Antiaris toxicaria* (NC_042884) was used as the outgroup. Subgeneric assignments were adopted following established taxonomic and molecular treatments (Berg, 1989; Rønsted et al., 2008; Cruaud et al., 2012). Sequences were aligned with MAFFT version 7.450 (Katoh & Standley, 2013), and the best-fit model (General Time Reversible (GTR)+F) was determined using ModelFinder in IQ-TREE version 2.0.3 (Minh et al., 2020). Maximum likelihood (ML) analysis was conducted with IQ-TREE using 10,000 bootstrap replicates, and Bayesian inference (BI) was performed with MrBayes version 3.2.7 (Ronquist & Huelsenbeck, 2003) *via* four parallel Markov chain Monte Carlo (MCMC) runs of 1,000,000 generations each, sampling every 500 generations and discarding the first 25% of samples as burn-in. Trees were visualized using FigTree version 1.4.4 (Drummond et al., 2012).

## Results

### General features

Complete chloroplast genomes for the seven *Ficus* species (*F. esquiroliana*, PQ526730; *F. pandurata*, PQ526731; *F. formosana*, PQ526732; *F. erecta*, PQ526733; *F*. *carica*, PQ526734; *F. hirta*, PQ526735; and *F. stenophylla*, PQ526736) were successfully assembled from Illumina HiSeq paired-end reads (150 bp) using NOVOPlasty software and deposited at National Center for Biotechnology Information (NCBI).

**Figure 1:**
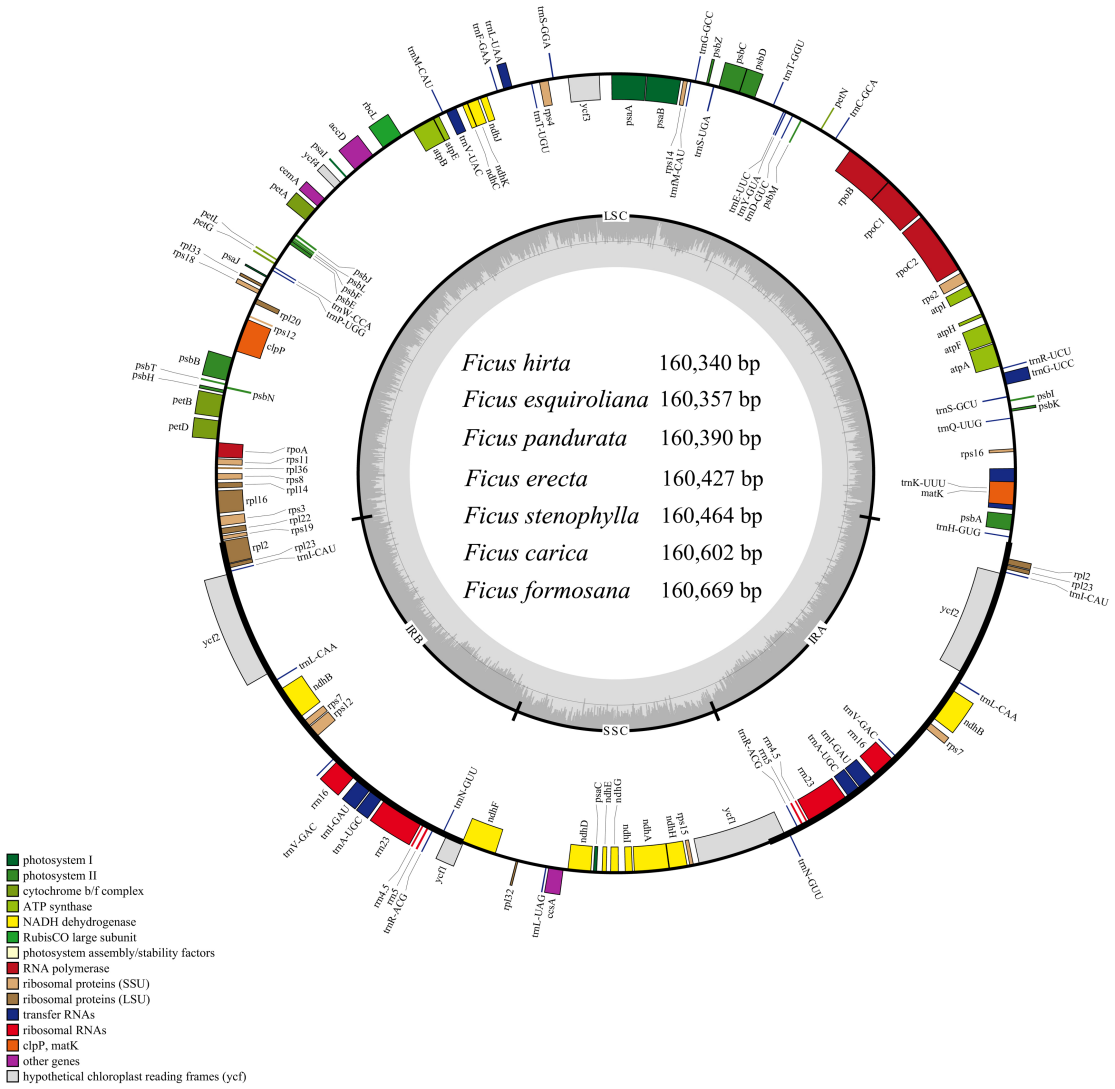
Gene map of the seven *Ficus* species cp genomes. The innermost shaded areas inside the inner circle correspond to the GC content in the cp genome. Genes in different functional groups are color coded. The boundaries of four regions (IRa, IRb, LSC, SSC) are noted in the inner circle.

The *Ficus* plastomes consistently exhibited a typical quadripartite structure with conserved gene content, position, and orientation (Fig. 1), and their sizes, GC contents, numbers of genes, and other information are shown in Table S3. The cp genomes of *Ficus* species ranged in length from 160,340 bp (*F. hirta*) to 160,669 bp (*F. formosana*). The GC content was 35.9%. The total number of annotated genes in *Ficus* plastomes was 130, comprising 85 (79+6) protein-coding genes (PCGs), 37 (30+7) tRNA genes, and 8 (4+4) rRNA genes. Within these genomes, 18 intron-containing genes were identified (six tRNA and 12 PCGs). Among these intron-containing genes, 15 contain one intron (*ndh*A, *ndh*B, *atp*F, *pet*B, *pet*D, *rpo*C1, *rps*16, *rpl*2, *rpl*16, *trn*A-UGC, *trn*G-UCC, *trn*I-GAU, *trn*K-UUU, *trn*L-UAA, and *trn*V-UAC), while the other three genes (*rps*12, *clp*P and *ycf*3) contain two introns (Table S4).

### Repeated sequences

A total of 506 complex repeat sequences, consisting of 210 interspersed repeats (64 forward, nine reverse, 134 palindrome, and three complementary) and 296 tandem repeats, were identified within plastome genomes using MISA, REPuter and Tandem Repeat Finder software, as described in the Material and Methods section (Fig. S1A). For interspersed repeats, the sequence length is mainly concentrated in 30–64 bp, regardless of the forward or palindrome repeats. As for the tandem repeats, most were in the range of 8–26 bp, and a four bp repeat was observed in *F. formosana* and *F. carica* within the *ycf*3 gene. These tandem repeats were mainly distributed in the non-coding LSC and SSC regions.

Microsatellites are small repeating units (one to six nucleotide) within a genome nucleotide sequence (Shukla et al., 2018). The high polymorphism rate of repeat sequences at the species level positions them as common molecular markers valuable for phylogenetic and population genetics studies (Zheng et al., 2020). An analysis of SSRs revealed a count ranging from 89 to 98 in the plastomes (Fig. S1B). The distribution of SSR types was predominantly mononucleotide repeats (59%), among which A/T repeats were the most numerous. Dinucleotides were the next most frequent at 22%, and tetranucleotides made up 9%, with other SSR types occurring at lower rates (Figs. S1C; S1D). This composition pattern suggests that mononucleotide repeats potentially play a greater role in genetic variability than other SSR types. Our finding that A/T repeats were most abundant is similar to reports from other studies (Munyao et al., 2020). Analysis of SSR locations further indicated that the majority were situated in non-coding, intergenic, and intron regions. These SSRs offer potential for developing specific markers crucial for systematic, evolutionary, and conservation studies in *Ficus*.

### Codon usage

**Figure 2:**
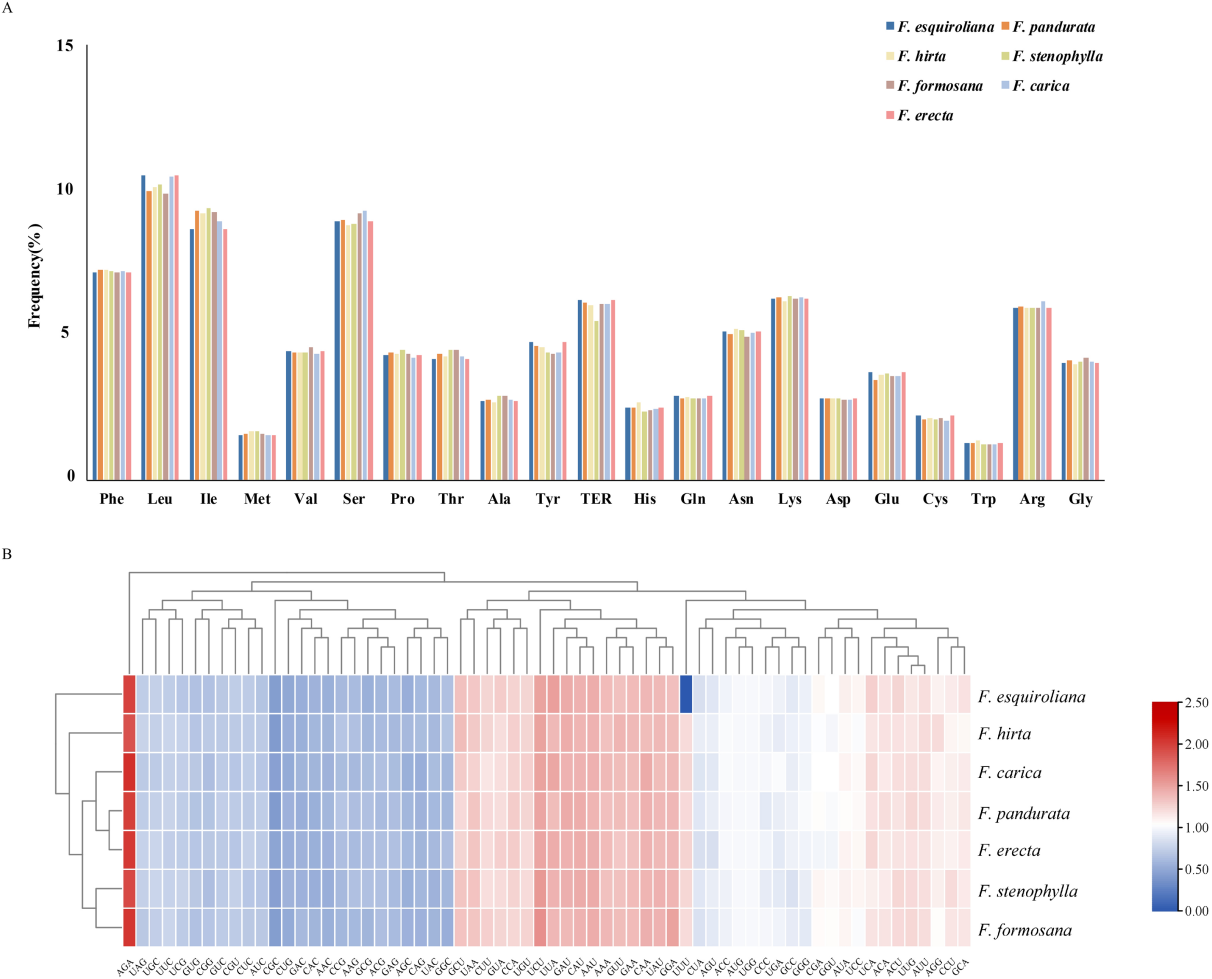
(A) Relative frequency of each amino acid (expressed as a percentage) in seven *Ficus* species (B) Heat map analysis for relative synonymous codon usage (RSCU) values of all protein-coding genes of seven complete chloroplast genomes in. Red and blue indicates higher and lower RSCU values, respectively.

The patterns of codon usage and nucleotide composition contribute to establishing a theoretical foundation for the genetic modification of cp genomes (Mazumdar et al., 2017). A comprehensive analysis was conducted on the codon usage frequency of protein-coding genes within the chloroplast genomes of seven assembled species of *Ficus*. The study revealed 64 distinct RSCU values within the plastomes of these *Ficus* species. These genomes contain between 53,455 and 53,556 codons, with *F. hirta* containing the fewest and *F. formosana* containing the most. Among all the codons, leucine (Leu) emerged as the most prevalent amino acid, with a frequency ranging from 9.7 to 10.49%. This was followed by isoleucine (Ile), with a frequency between 8.64% and 9.35%. Conversely, tryptophan (Trp) was the least abundant, with a frequency of 1.21–1.35% (Fig. 2A). Methionine and tryptophan are each encoded by a single codon (ATG and TGG, respectively); therefore their RSCU values are 1.00 by definition and RSCU is not informative for assessing synonymous codon bias for these amino acids. Thirty codons were identified with an RSCU value greater than 1. Among them, except for UUG (Leu) and AGG (Arg), all codons ended with A or U(T) nucleotides (Fig. 2B). This observation suggested a preference for A and T as the terminal bases in codons.

### Comparative analysis between seven *Ficus* species

**Figure 3:**
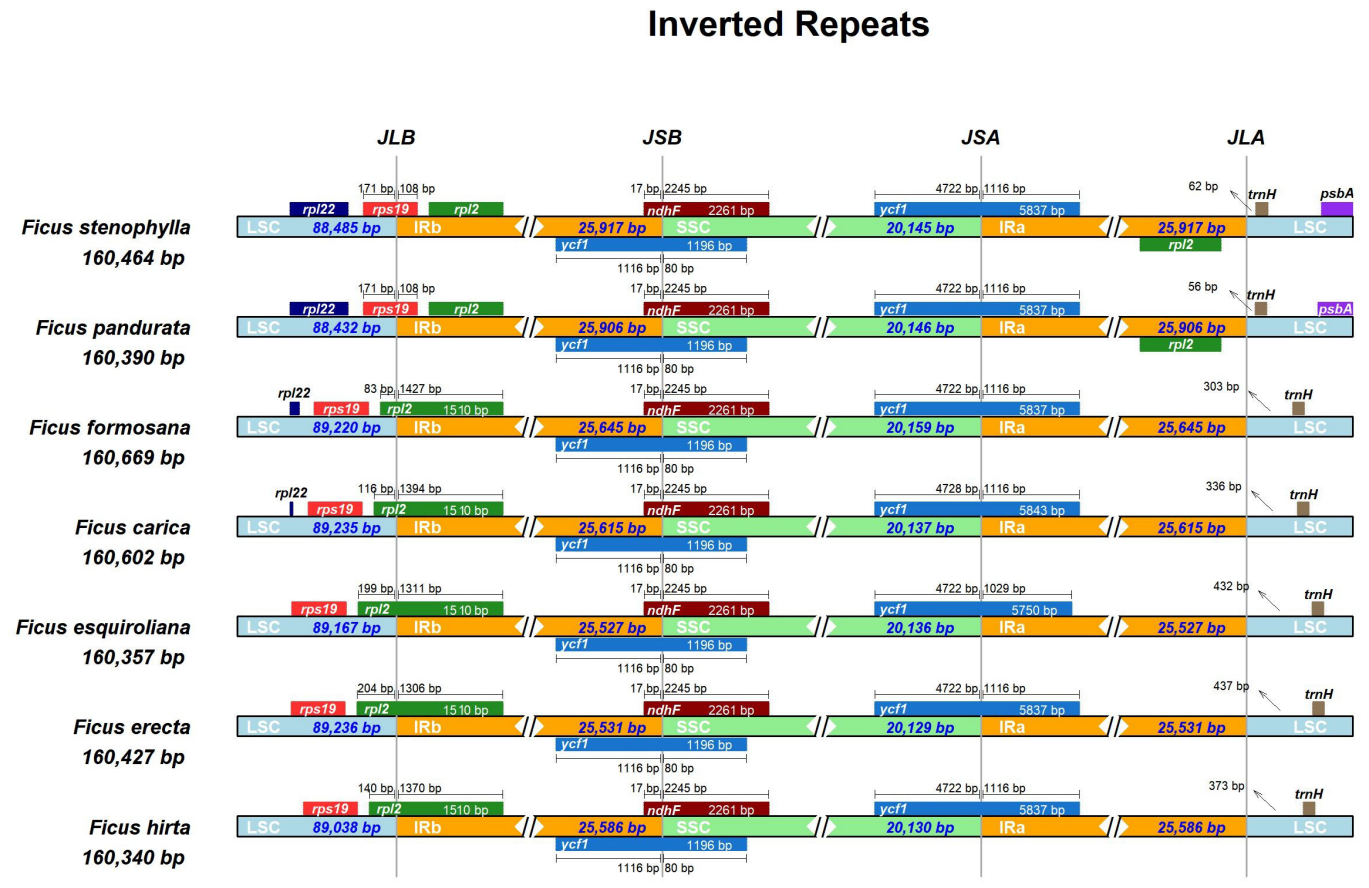
Comparison of the borders of LSC, SSC, and IR regions among chloroplast genomes of *Ficus*. The number of base pairs (bp) represents the distance from the boundary to the end of the gene. JL, junction between the large single-copy region (LSC) and the inverted repeat (IR); JS, junction between the Inverted Repeat (IR) and the small single-copy region (SSC).

The expansion and contraction of the IR region are major driving forces in the evolution of land plants (Kode et al., 2005; Raubeson et al., 2007; Yao et al., 2015; Abdullah Mehmood et al., 2020). In this study, we compared the positions of the LSC/IR junction (JL) and the IR/SSC junction (JS) across seven assembled *Ficus* plastomes (Fig. 3). The lengths of the IR regions were relatively uniform, ranging from 25,527 to 25,917 bp, indicating high conservation with minimal contraction/expansion across the seven *Ficus* plastomes. The JL (IR-LSC: *rpl*2 **&** *rps*19) boundary showed high similarity in seven *Ficus* plastomes, except for *F. pandurata* and *F. stenophylla*. At the IR-LSC boundary, the *rps* 19 gene crossed into the IR region by approximately 108 bp in *F. pandurata* and *F. stenophylla*, while it remained entirely in the LSC region in the other five species. The JS (IR-SSC: *ycf*1 **&** *ndh*F) boundaries were also highly similar in *Ficus* plastomes. The *ycf*1 gene crossed the IR-SSC border and extended into the IR region at approximately 1,116 bp. Concurrently, the *ndh* F gene consistently extended into the IRB region by 17 bp in all species, with the remainder of the gene located in the SSC. At the JSA (SSC-IRA) boundary, the *ycf*1 gene partially extended into the SSC region for all species, with its segment in SSC measuring 4,722 bp in six species and 4,728 bp in *F. esquiroliana*. At the JLA (IRA/LSC) boundary, the length of the intergenic spacer between the boundary and the *trn*H gene, which is located in the LSC region, exhibits significant variation among different species, ranging from 56 bp in *F. pandurata* to 437 bp in *F. erecta*.

**Figure 4:**
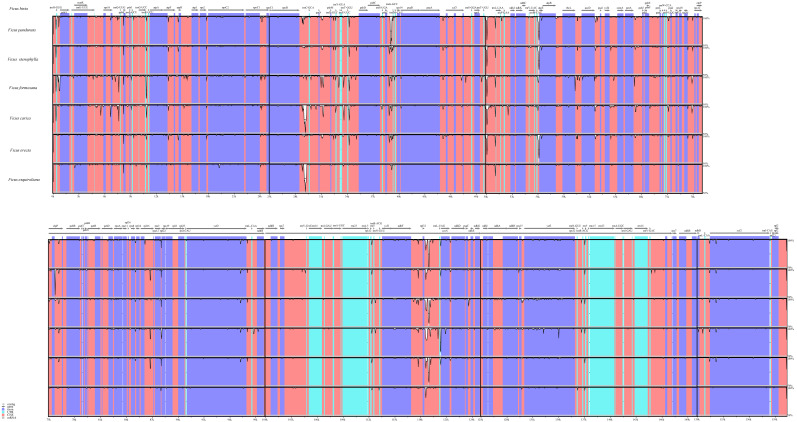
Visualized alignment of the *Ficus* chloroplast genome sequences with annotations using mVISTA. Each horizontal lane shows the graph for the sequence pairwise identity with *F. hirta* as reference. The *x*-axis represents the base sequence of the alignment and the *y*-axis represents the pairwise percent identity within 50–100%. Gray arrows represent the genes and their orientations. Dark-blue boxes represent exon regions; light-blue boxes represent untranslated regions; red boxes represent conserved non-coding sequence (CNS) regions.

We also analyzed the differences in the cp genomes among the seven species through global sequence alignment (Fig. 4). Using mVISTA for global sequence alignment, with *F. hirta* sequence and annotation file as references, we observed variations across different regions among the seven species. The results indicated that the seven chloroplast genomes are highly conserved. Furthermore, the alignment revealed higher sequence conservation in coding regions compared to non-coding regions, and in inverted repeat regions relative to single-copy regions (Fig. 4).

**Figure 5:**
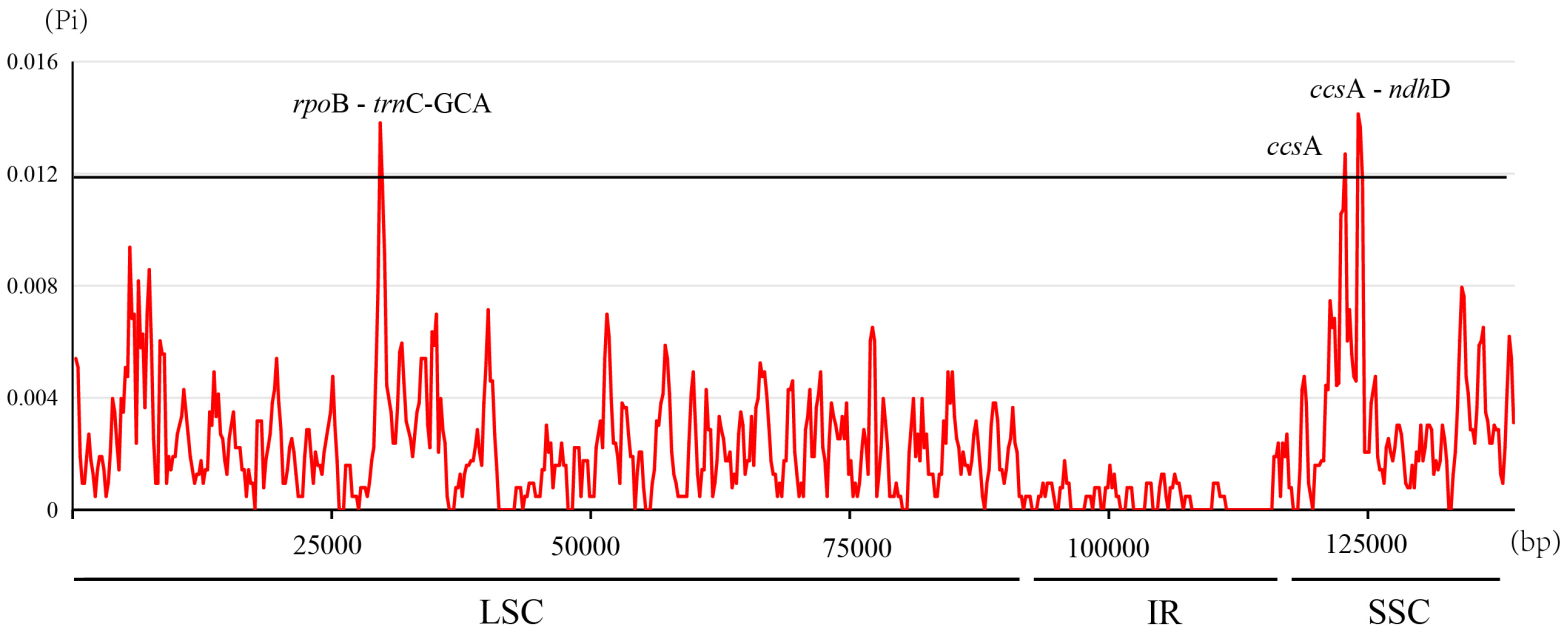
Nucleotide diversity in the *Ficus* chloroplast genomes.

The nucleotide diversity (Pi, *π*) within *Ficus* plastomes ranged from 0 to 0.0141, with a mean value of 0.0022. Inverted repeat (IR) regions exhibited low nucleotide polymorphism, and the majority of variations were localized to the large single-copy (LSC) and small single-copy (SSC) regions (Fig. 5). While protein-coding regions demonstrated overall conservation across these plastomes, three gene regions (*ccs*A, *ccs*A - *ndh*D, and *rpo*B - *trn*C-GCA) displayed significantly elevated Pi values (>0.012). Notably, *ccs*A exhibited the highest divergence, reaching a Pi value of 0.0141 (Fig. 5). These polymorphic loci serve as promising candidate barcode sequences for phylogenetic inference and population genetic studies within *Ficus*.

### Phylogenetic analysis

**Figure 6:**
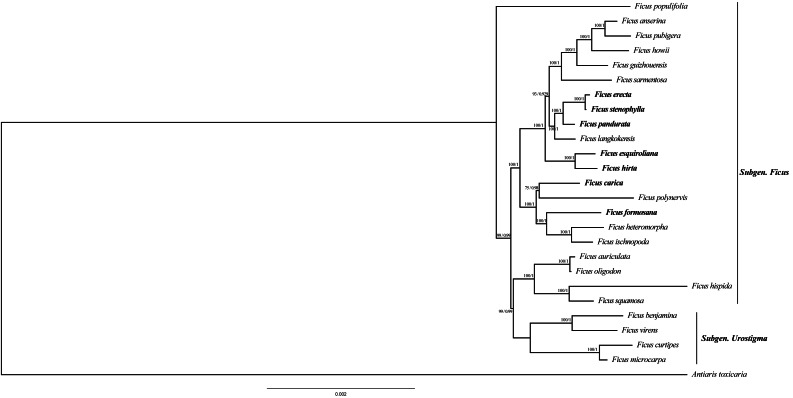
Maximum likelihood tree and Bayesian tree were constructed based on CDS data partitions of 26 species chloroplast genomes. The number on the branches as Bayesian inference posterior probability/maximum likelihood bootstrap support values. Branch lengths represent the expected number of substitutions per site (scale bar = 0.002 substitutions per site).

To ascertain the phylogenetic relationships among *Ficus* species, we employed 79 PCGs from 26 species to reconstruct phylogenetic trees. The results indicate that the topology generated by distance estimation using the best fit model (GTR+F) is consistent under two different reconstruction methods (ML and BI) (Fig. 6). All *Ficus* species formed a monophyletic clade, separate from the outgroup *Antiaris toxicaria*. Notably, all seven newly sequenced plastomes in this study are assigned to *Subgen. Ficus* (Table S2) and are distributed into two strongly supported subclades (Fig. 6). Within one clade, *F. hirta*, *F. esquiroliana*, *F. erecta*, *F. stenophylla*, *F. pandurata*, and *F. langkokensis* clustered together with 100% bootstrap support, indicating a close relationship among these species. Within the other clade, *F. carica*, *F. polynervis*, *F. formosana F. heteromorpha*, and *F. ischnopoda* clustered together, suggesting a close relationship. All branches had support rates above 70%.

## Discussion

This study presents the complete chloroplast genomes of seven *Ficus* species, contributing to the understanding of cp genome evolution, sequence divergence, and phylogenetic relationships within the genus. The plastomes exhibited a typical quadripartite structure with highly conserved gene content, order, and orientation, which is consistent with previous reports of other members of Moraceae and angiosperms more broadly (Jansen et al., 2005; Zhang et al., 2022a; Zhang et al., 2022b). The minimal variation in overall genome sizes (160,340–160,669 bp) and the uniform GC content of approximately 35.9% further reflect the characteristic slow structural evolution and high conservation of chloroplast genomes in land plants.

### Repeat Sequences and SSRs

We found a substantial number of complex repeat sequences and simple sequence repeats (SSRs) in the *Ficus* plastomes. The predominance of mononucleotide (A/T-rich) SSRs is a common feature in angiosperm chloroplast genomes (Munyao et al., 2020; Provan, Powell & Hollingsworth, 2001), likely reflecting underlying mutational biases and relaxed selection in non-coding regions. These identified SSRs and other repeat motifs represent valuable resources for developing high-resolution genetic markers, which can be applied in population genetics, phylogeography, and species identification within *Ficus* (Zhang et al., 2022a; Zhang et al., 2022b). The observed distribution of SSRs, mainly in non-coding regions, also supports their utility as informative hotspots for molecular marker development.

### Codon usage bias

Analysis of codon usage revealed a strong bias toward codons ending in A or T, consistent with the compositional bias in angiosperm chloroplast genomes (Mazumdar et al., 2017). This bias may reflect selection for translational efficiency, mutational pressures, or a combination of both. The predominance of leucine and isoleucine codons, and the low frequency of tryptophan, is in line with findings from other plant cp genomes (Morton, 1998). Such codon usage patterns provide fundamental information for future studies of plastome gene expression and for potential transplastomic engineering in *Ficus*.

### IR boundary dynamics

The expansion and contraction of inverted repeat (IR) regions are known to contribute to size variation and evolutionary novelty in plastid genomes (Wang et al., 2008; Xiong et al., 2009). Our comparative analysis of the seven *Ficus* chloroplast genomes revealed a generally conserved quadripartite structure, with IR region lengths ranging from 25,527 bp to 25,917 bp, indicating relative stability within this genus. However, detailed examination of the LSC/IR and IR/SSC junctions (Fig. 3) unveiled minor yet discernible variations, reflecting dynamic micro-evolutionary shifts. The presence of partial *rps*19 and *rpl*2 duplications at the respective boundaries suggests that both lineage-specific and potentially ongoing border shifts may occur within *Ficus*. However, the relatively limited IR boundary variation suggests a largely stable cp genome structure in this genus, compared to the more dramatic IR dynamics seen in some other angiosperms.

### Potential molecular markers in *Ficus*

Global sequence alignment and nucleotide diversity (Pi) analyses demonstrated that, as expected, the IR regions were the most conserved while the single-copy regions especially non-coding and intergenic spacers exhibited higher sequence divergence. The identification of three highly variable regions (*ccs*A, *ccs*A - *ndh*D, and *rpo*B - *trn*C-GCA) with elevated Pi values highlights their potential as candidate DNA barcodes for *Ficus* systematics and population studies. These hypervariable loci may facilitate more precise species delimitation and phylogeographic analyses, potentially complementing or providing higher resolution than existing universal barcode regions such as *mat*K and *rbc*L (CBOL Plant Working Group, 2009).

### Phylogenetic analysis in *Ficus*

Phylogenetic reconstruction based on a concatenated alignment of 79 chloroplast protein-coding genes robustly resolved relationships among the seven newly sequenced *Ficus* species nested within a monophyletic *Ficus* clade (26 taxa sampled). The recovered topology, which is consistent between ML and BI analyses and shows high support across nodes, identifies two major subclades that correspond well with previously recognized divisions based on nuclear and plastid markers (Rønsted et al., 2008). In our sampling most newly sequenced taxa are assigned to *Subgen. Ficus* and are distributed across two well-supported subclades; a small set of *Subgen. Urostigma* taxa were included to provide taxonomic context.

While plastome-scale data markedly improve resolution relative to single- or few-locus approaches and can help resolve recent radiations, chloroplast genomes represent a single, typically uniparentally inherited locus and therefore may not fully capture complex reticulate histories (*e.g.*, hybridization, introgression, incomplete lineage sorting) that are likely in diverse genera such as *Ficus*. Moreover, our sampling-seven new plastomes within a genus of >800 species-remains limited. To more rigorously assess subgeneric monophyly and the deeper relationships within *Ficus* will require denser taxon sampling across recognized subgenera and integration of nuclear (and mitochondrial) genomic datasets. Finally, organellar complementarity deserves mention. While chloroplast genomes provide a relatively straightforward single-locus phylogenomic perspective, mitochondrial genomes and nuclear datasets provide independent and complementary histories. The recent publication of the complete mitochondrial genome of *F. hirta* (Deng & Cai, 2025) highlights an opportunity for combined organellar analyses; for example, comparisons of shared repeats, transferred sequences, or conflicting topologies between organellar genomes may help detect events such as introgression or assembly artifacts. We therefore encourage future work that integrates chloroplast, mitochondrial and nuclear genomic data, coupled with denser taxon and geographic sampling, to provide a more complete picture of *Ficus* evolutionary history.

## Conclusions

We present seven additional complete chloroplast genomes for *Ficus* species, expanding available organellar genomic resources for the genus. Comparative analyses revealed conserved quadripartite genome structure, lineage-specific minor IR boundary variation, abundant A/T-biased SSRs concentrated in noncoding regions, codon usage bias toward A/T endings, and several hypervariable regions (*ccs*A, *ccs*A - *ndh*D, *rpo*B - *trn*C-GCA) that are promising candidate loci for species delimitation and population studies. These plastome data, when integrated with denser taxon sampling and nuclear/mitochondrial datasets, will aid future studies of *Ficus* systematics, biogeography, and conservation.

## Supplemental Information

10.7717/peerj.20531/supp-1 Supplemental Information 1 Raw data

10.7717/peerj.20531/supp-2 Supplemental Information 2 Quantitative analysis of various repeat types in *Ficus* chloroplast genomes (A) The number of Dispersed repeat and Tandem repeat; (B) Number of various SSR repeat types; (C) The proportion of SSR repeat types across all seven species; (D) Number of SSRs in each species.

10.7717/peerj.20531/supp-3 Supplemental Information 3 Sampling information for *Ficus* species in this study

10.7717/peerj.20531/supp-4 Supplemental Information 4 List of species used for phylogenetic tree construction

10.7717/peerj.20531/supp-5 Supplemental Information 5 Summary of characteristics of *Ficus* chloroplast genomes

10.7717/peerj.20531/supp-6 Supplemental Information 6 List of annotated genes in *Ficus* chloroplast genome

## Abbreviations

IR: Inverted repeat
LSC: Large single copy
SSC: Small single copy
rRNA: Ribosomal rNA
tRNA: Transfer rNA
CTAB: Cetyltrimethylammonium bromide
PCR: Polymerase chain reaction
SSR: Simple sequence repeat
BI: Bayesian inference
ML: Maximum likelihood
BS: Branch support

## Additional Information and Declarations

### Data Availability

The following information was supplied regarding data availability: The raw data is available in the Supplemental Files. The raw sequence data are available at NCBI: PQ526730–PQ526736.

## References

[ref-1] Abdullah Mehmood F, Shahzadi I, Waseem S, Mirza B, Ahmed I, Waheed MT (2020). Chloroplast genome of *Hibiscus rosa-sinensis* (Malvaceae): comparative analyses and identification of mutational hotspots. Genomics.

[ref-2] Amiryousefi A, Hyvönen J, Poczai P (2018). IRscope: an online program to visualize the junction sites of chloroplast genomes. Bioinformatics.

[ref-3] Beier S, Thiel T, Münch T, Scholz U, Mascher M (2017). MISA-web: a web server for microsatellite prediction. Bioinformatics.

[ref-4] Benson G (1999). Tandem repeats finder: a program to analyze DNA sequences. Nucleic Acids Research.

[ref-5] Berg CC (1989). Classification and distribution of *Ficus*. Cellular & Molecular Life Sciences.

[ref-6] Bolger AM, Lohse M, Usadel B (2014). Trimmomatic: a flexible trimmer for illumina sequence data. Bioinformatics.

[ref-7] CBOL Plant Working Group (2009). A DNA barcode for land plants. Proceedings of the National Academy of Sciences of the United States of America.

[ref-8] Chen CJ, Chen H, Zhang Y, Thomas HR, Frank MH, He YH, Xia R (2020). TBtools: an integrative toolkit developed for interactive analyses of big biological data. Molecular Plant.

[ref-9] Cruaud A, Nina R, Chantarasuwan B, Chou LS, Clement WL, Couloux A, Cousins B, Genson G, Harrison RD, Hanson PE, Hossaert-McKey M, Jabbour-Zahab R, Jousselin E, Kerdelhué C, Kjellberg F, Lopez-Vaamonde C, Peebles J, Peng YQ, Pereira RAS, Schramm T, Ubaidillah R, Noort S, Weiblen GD, Yang DR, Yodpinyanee A, Libeskind-Hadas R, Cook JM, Rasplus JY, Savolainen V (2012). An extreme case of plant-insect codiversification: figs and fig-pollinating wasps. Systematic Biology.

[ref-10] Daniell H, Lin CS, Yu M, Chang WJ (2016). Chloroplast genomes: diversity, evolution, and applications in genetic engineering. Genome Biology.

[ref-11] Deng WD, Cai XY (2025). Complete mitochondrial genome of *Ficus hirta* and its comparative analysis. Frontiers in Genetics.

[ref-12] Devi R, Manjula BL, Kumar M, Kumar S, Marndi S (2022). Food and medicinal values of some *Ficus* species. Medico-Biowealth of India.

[ref-13] Dierckxsens N, Mardulyn P, Smits G (2017). NOVOPlasty: *de novo* assembly of organelle genomes from whole genome data. Nucleic Acids Research.

[ref-14] Doyle JJ, Doyle JL (1987). A rapid DNA isolation procedure for small quantities of fresh leaf tissue. Phytochemical Bulletin.

[ref-15] Drummond AJ, Suchard MA, Xie D, Rambaut A (2012). Bayesian phylogenetics with BEAUti and the BEAST 1.7. Molecular Biology and Evolution.

[ref-16] Duan N, Deng LL, Zhang Y, Shi YC, Liu BB (2022). Comparative and phylogenetic analysis based on chloroplast genome of *Heteroplexis* (Compositae), a protected rare genus. BMC Plant Biology.

[ref-17] Frazer KA, Pachter L, Poliakov A, Rubin EM, Dubchak I (2004). VISTA: computational tools for comparative genomics. Nucleic Acids Research.

[ref-18] Greiner S, Lehwark P, Bock R (2019). OrganellarGenomeDRAW (OGDRAW) version 1.3.1: expanded toolkit for the graphical visualization of organellar genomes. Nucleic Acids Research.

[ref-19] Huang DI, Cronk QCB (2015). Plann: a command-line application for annotating plastome sequences. Applications in Plant Sciences.

[ref-20] Huang YY, Li J, Yang ZR, An WL, Xie CZ, Liu SS, Zheng XS (2022). Comprehensive analysis of complete chloroplast genome and phylogenetic aspects of ten *Ficus* species. BMC Plant Biology.

[ref-21] Jansen RK, Raubeson LA, Boore JL, Depamphilis CW, Chumley TW, Haberle RC, Wyman SK, Alverson AJ, Peery R, Herman SJ, Fourcade HM, Kuehl JV, McNeal JR, Mack JL, Cui LY (2005). Methods for obtaining and analyzing whole chloroplast genome sequences. Methods in Enzymology.

[ref-22] Katoh K, Standley DM (2013). MAFFT multiple sequence alignment software version 7: improvements in performance and usability. Molecular Biology and Evolution.

[ref-23] Kearse M, Moir R, Wilson A, Stones-Havas S, Cheung M, Sturrock S, Buxton S, Cooper A, Markowitz S, Duran C, Thierer T, Ashton B, Meintjes P, Drummond A (2012). Geneious basic: an integrated and extendable desktop software platform for the organization and analysis of sequence data. Bioinformatics.

[ref-24] Kode V, Mudd EA, Iamtham S, Day A (2005). The *tobacco* plastid *acc* D gene is essential and is required for leaf development. The Plant Journal.

[ref-25] Kurtz S, Choudhuri JV, Ohlebusch E, Schleiermacher C, Stoye J, Robert G (2001). REPuter: the manifold applications of repeat analysis on a genomic scale. Nucleic Acids Research.

[ref-26] Mazumdar P, Binti Othman RY, Mebus K, Ramakrishnan N, Harikrishna JN (2017). Codon usage and codon pair patterns in non-grass monocot genomes. Annals of Botany.

[ref-27] Minh BQ, Schmidt HA, Chernomor O, Schrempf D, Woodhams MD, Haeseler AV, Lanfear R (2020). IQ-TREE 2: new models and efficient methods for phylogenetic inference in the genomic era. Molecular Biology and Evolution.

[ref-28] Morton BR (1998). Selection on the codon bias of chloroplast and cyanelle genes in different plant and algal lineages. Journal of Molecular Evolution.

[ref-29] Munyao JN, Dong X, Yang JX, Mbandi EM, Wanga VC, Oulo MA, Saina JK, Musili PM, Hu GW (2020). Complete chloroplast genomes of *Chlorophytum comosum* and *Chlorophytum gallabatense*: genome structures, comparative and phylogenetic analysis. Plants.

[ref-30] Provan J, Powell W, Hollingsworth PM (2001). Chloroplast microsatellites: new tools for studies in plant ecology and evolution. Trends in Ecology & Evolution.

[ref-31] Raji IA, Downs CT (2021). Ficus-frugivore interactions, especially in areas of land-use change, in Africa: a systematic review. Acta Oecologica.

[ref-32] Raubeson LA, Peery R, Chumley TW, Dziubek C, Fourcade HM, Boore JL, Jansen RK (2007). Comparative chloroplast genomics: analyses including new sequences from the angiosperms *Nuphar advena* and *Ranunculus macranthu*s. BMC Genomics.

[ref-33] Ronquist F, Huelsenbeck JP (2003). MrBayes 3: bayesian phylogenetic inference under mixed models. Bioinformatics.

[ref-34] Rønsted N, Weiblen GD, Clement WL, Zerega NJC, Savolainen V (2008). Reconstructing the phylogeny of figs (*Ficus*, Moraceae) to reveal the history of the fig pollination mutualism. Symbiosis.

[ref-35] Rozas J, Ferrer AM, Sánchez-DelBarrio JC, Guirao-Rico S, Librado P, Ramos-Onsins SE, Sánchez-Gracia A (2017). DnaSP 6: dNA sequence polymorphism analysis of large data sets. Molecular Biology and Evolution.

[ref-36] Sharp PM, Li WH (1986). An evolutionary perspective on synonymous codon usage in unicellular organisms. Journal of Molecular Evolution.

[ref-37] Shukla N, Kuntal H, Shanker A, Sharma SN (2018). Mining and analysis of simple sequence repeats in the chloroplast genomes of genus *Vigna*. Biotechnology Research and Innovation.

[ref-38] Vu TTT, Vu LTK, Le LT, Lo TTM, Chu MH (2023). Analysis of the chloroplast genome of *Ficus simplicissima* lour collected in Vietnam and proposed barcodes for identifying *Ficus* plants. Current Issues in Molecular Biology.

[ref-39] Wang RJ, Cheng CL, Chang CC, Wu CL, Su TM, Chaw SM (2008). Dynamics and evolution of the inverted repeat-large single copy junctions in the chloroplast genomes of monocots. BMC Evolutionary Biology.

[ref-40] Xia X, Peng JY, Yang L, Zhao XL, Duan A, Wang DW (2022). Comparative analysis of the complete chloroplast genomes of eight *Ficus* species and insights into the phylogenetic relationships of *Ficus*. Life.

[ref-41] Xiong AS, Peng RH, Zhuang J, Gao F, Zhu B, Fu XY, Xue Y, Jin XF, Tian YS, Zhao W, Yao QH (2009). Gene duplication, transfer, and evolution in the chloroplast genome. Biotechnology Advances.

[ref-42] Yao XH, Tang P, Li ZZ, Li DW, Liu YF, Huang HW (2015). The first complete chloroplast genome sequences in Actinidiaceae: genome structure and comparative analysis. PLOS ONE.

[ref-43] Zeng QW, Chen M, Wang SC, Xu XX, Li T, Xiang ZH, He NJ (2022). Comparative and phylogenetic analyses of the chloroplast genome reveal the taxonomy of the *Morus* genus. Frontiers in Plant Science.

[ref-44] Zhang XT, Wang G, Zhang SC, Cheng S, Wang YB, Wen P, Ma XK, Shi Y, Qi R, Yang Y, Liao ZY, Lin J, Xu XM, Chen XQ, Xu SD, Deng F, Zhao LH, Lee LY, Wang R, Chen XY, Lin YR, Zhang JS, Tang HB, Chen J, Ming R (2020). Genomes of the Banyan tree and pollinator wasp provide insights into fig-wasp coevolution. Cell.

[ref-45] Zhang ZR, Yang X, Li WY, Peng YQ, Gao J (2022b). Comparative chloroplast genome analysis of *Ficus* (Moraceae): insight into adaptive evolution and mutational hotspot regions. Frontiers in Plant Science.

[ref-46] Zhang Z, Zhang DS, Zou L, Yao CY (2022a). Comparison of chloroplast genomes and phylogenomics in the *Ficus sarmentosa* complex (Moraceae). PLOS ONE.

[ref-47] Zheng G, Wei LL, Ma L, Wu ZQ, Gu CH, Chen K (2020). Comparative analyses of chloroplast genomes from 13 *Lagerstroemia* (Lythraceae) species: identification of highly divergent regions and inference of phylogenetic relationships. Plant Molecular Biology.

