# Supplementary figures and images for: Comparative genomics and phylogenetic analysis of seven Ficus species based on chloroplast genomes
Source: PeerJ. 2026 Jan 7;14:e20531. doi: 10.7717/peerj.20531 (PMC12790284; doi:10.7717/peerj.20531)

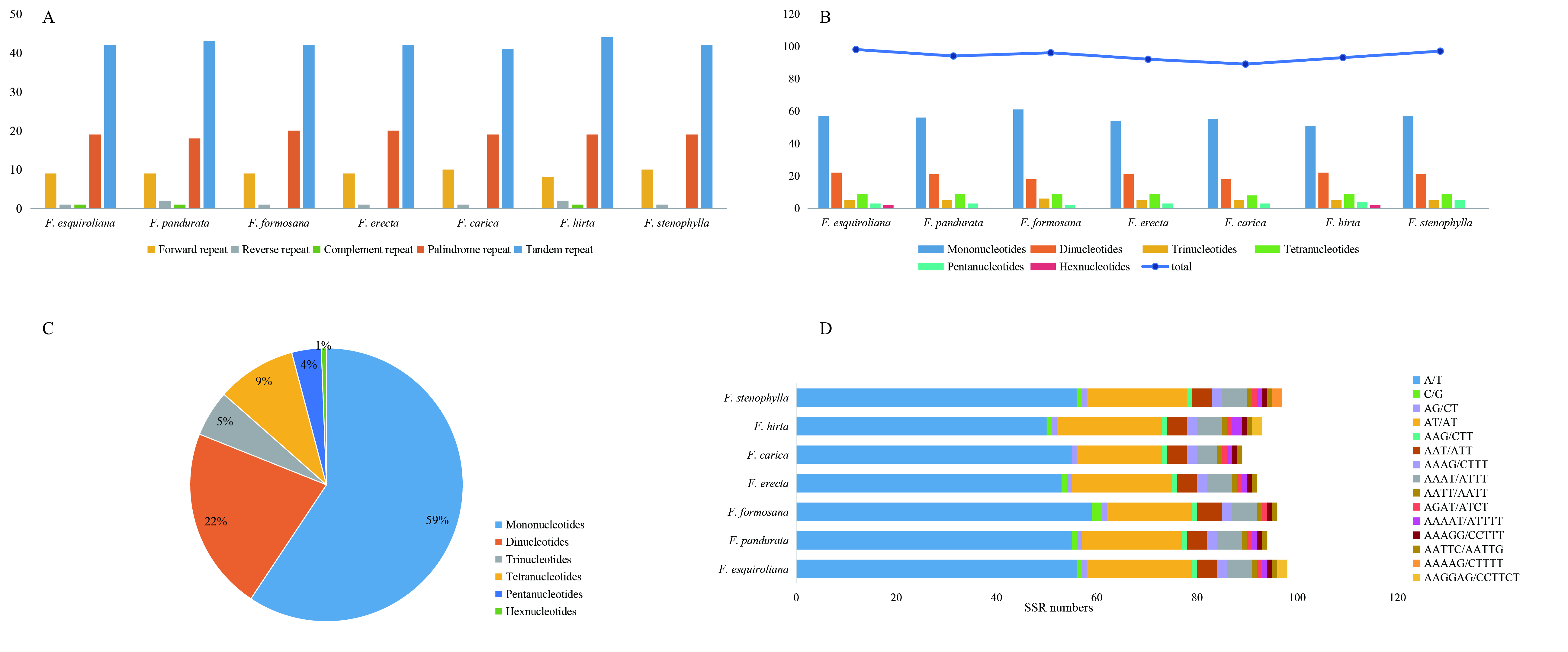

Supplement: Supplemental Information 2 — (A) The number of Dispersed repeat and Tandem repeat; (B) Number of various SSR repeat types; (C) The proportion of SSR repeat types across all seven species; (D) Number of SSRs in each species. [file peerj-14-20531-s002.jpg]
