# Supplementary material for: Comparative genomics and phylogenetic analysis of seven Ficus species based on chloroplast genomes
Source: PeerJ. 2026 Jan 7;14:e20531. doi: 10.7717/peerj.20531 (PMC12790284; doi:10.7717/peerj.20531)
Supplement: Supplemental Information 3 [file peerj-14-20531-s003.docx]

| Table S1 Sampling information for Ficus species in this study. | | | | | | |
| --- | --- | --- | --- | --- | --- | --- |
| **Species** | **Locality** | **longitude** | **latitude** | **Elevation(m)** | **Voucher** | **Source** |
| *F. esquiroliana* | Yanshan District, Guilin City | 110.3 | 25.07 | 264 | Shi20240617_hmr | Guangxi Institute of Botany |
| *F. pandurata* | Shaxian District, Sanming City | 117.85 | 26.37 | 165 | Shi20240617_qyr | Guangxi Institute of Botany |
| *F. formosana* | Shaxian District, Sanming City | 117.85 | 26.37 | 165 | Shi20240617_twr | Guangxi Institute of Botany |
| *F. erecta* | Lingui District, Guilin City | 110.09 | 25.45 | 320 | Shi20240617_txg | Guangxi Institute of Botany |
| *F. carica* | Yanshan District, Guilin City | 110.3 | 25.07 | 264 | Shi20240617_whg | Guangxi Institute of Botany |
| *F. hirta* | Caiwan Town, Quanzhou County | 111.03 | 26.2 | 260 | Shi20240617_wzmt | Guangxi Institute of Botany |
| *F. stenophylla* | Shaxian District, Sanming City | 117.85 | 26.37 | 165 | Shi20240617_zyr | Guangxi Institute of Botany |
