# Supplementary material for: Comparative genomics and phylogenetic analysis of seven Ficus species based on chloroplast genomes
Source: PeerJ. 2026 Jan 7;14:e20531. doi: 10.7717/peerj.20531 (PMC12790284; doi:10.7717/peerj.20531)
Supplement: Supplemental Information 4 [file peerj-14-20531-s004.docx]

| Table S2 List of species used for phylogenetic tree construction. | | | | | |
| --- | --- | --- | --- | --- | --- |
| Family | Tribe | Genus | Subgen | Spcies | GeneBank accession |
| Moraceae | Ficeae | *Ficus* | *Ficus* | *Ficus esquiroliana* | PQ526730 |
| Moraceae | Ficeae | *Ficus* | *Ficus* | *Ficus pandurata* | PQ526731 |
| Moraceae | Ficeae | *Ficus* | *Ficus* | *Ficus formosana* | PQ526732 |
| Moraceae | Ficeae | *Ficus* | *Ficus* | *Ficus erecta* | PQ526733 |
| Moraceae | Ficeae | *Ficus* | *Ficus* | *Ficus carica* | PQ526734 |
| Moraceae | Ficeae | *Ficus* | *Ficus* | *Ficus hirta* | PQ526735 |
| Moraceae | Ficeae | *Ficus* | *Ficus* | *Ficus stenophylla* | PQ526736 |
| Moraceae | Ficeae | *Ficus* | *Ficus* | *Ficus populifolia* | NC_069638 |
| Moraceae | Ficeae | *Ficus* | *Ficus* | *Ficus anserina* | NC_069046 |
| Moraceae | Ficeae | *Ficus* | *Ficus* | *Ficus pubigera* | NC_069049 |
| Moraceae | Ficeae | *Ficus* | *Ficus* | *Ficus howii* | NC_069045 |
| Moraceae | Ficeae | *Ficus* | *Ficus* | *Ficus guizhouensis* | NC_069047 |
| Moraceae | Ficeae | *Ficus* | *Ficus* | *Ficus sarmentosa* | NC_061976 |
| Moraceae | Ficeae | *Ficus* | *Ficus* | *Ficus longkokensis* | NC_066476 |
| Moraceae | Ficeae | *Ficus* | *Ficus* | *Ficus polynervis* | NC_069048 |
| Moraceae | Ficeae | *Ficus* | *Ficus* | *Ficus heteromorpha* | NC_053835 |
| Moraceae | Ficeae | *Ficus* | *Ficus* | *Ficus ischnopoda* | NC_066475 |
| Moraceae | Ficeae | *Ficus* | *Ficus* | *Ficus auriculata* | OK078618 |
| Moraceae | Ficeae | *Ficus* | *Ficus* | *Ficus oligodon* | OK078619 |
| Moraceae | Ficeae | *Ficus* | *Ficus* | *Ficus hispida* | OQ220336 |
| Moraceae | Ficeae | *Ficus* | *Ficus* | *Ficus squamosa* | OK077764 |
| Moraceae | Ficeae | *Ficus* | *Urostigma* | *Ficus benjamina* | NC_053836 |
| Moraceae | Ficeae | *Ficus* | *Urostigma* | *Ficus virens* | NC_053832 |
| Moraceae | Ficeae | *Ficus* | *Urostigma* | *Ficus curtipes* | NC_053833 |
| Moraceae | Ficeae | *Ficus* | *Urostigma* | *Ficus microcarpa* | MW887640 |
| Moraceae | Olmedieae | *Antiaris* |  | *Antiaris toxicaria* | NC_042884 |
