# Supplementary material for: Comparative genomics and phylogenetic analysis of seven Ficus species based on chloroplast genomes
Source: PeerJ. 2026 Jan 7;14:e20531. doi: 10.7717/peerj.20531 (PMC12790284; doi:10.7717/peerj.20531)
Supplement: Supplemental Information 5 [file peerj-14-20531-s005.docx]

| Table S3 Summary of characteristics of *Ficus* chloroplast genomes. | | | | | | | |
| --- | --- | --- | --- | --- | --- | --- | --- |
| Spcies | *F. esquiroliana* | *F. pandurata* | *F. formosana* | *F. erecta* | *F. carica* | *F. hirta* | *F. stenophylla* |
| size(bp) | 160357 | 160390 | 160669 | 160427 | 160602 | 160340 | 160464 |
| LSC(bp) | 89167 | 88432 | 89220 | 89236 | 89235 | 89038 | 88485 |
| SSC(bp) | 20136 | 20146 | 20159 | 20129 | 20137 | 20130 | 20145 |
| IRs(bp) | 25527 | 25906 | 25645 | 25531 | 25615 | 25586 | 25917 |
| Total genes | 130 | 130 | 130 | 130 | 130 | 130 | 130 |
| Protein-coding genes | 85 | 85 | 85 | 85 | 85 | 85 | 85 |
| tRNA genes | 37 | 37 | 37 | 37 | 37 | 37 | 37 |
| rRNA genes | 8 | 8 | 8 | 8 | 8 | 8 | 8 |
| GC content | 35.90% | 35.90% | 35.90% | 35.90% | 35.90% | 35.90% | 35.90% |
| Protein-coding region(%) | 49.64% | 49.94% | 49.56% | 49.19% | 43.99% | 44.16% | 49.91% |
| LSC, large single copy; SSC, small single copy; IR, inverted repeat. | | | | | |  |  |
