# Supplementary material for: Comparative genomics and phylogenetic analysis of seven Ficus species based on chloroplast genomes
Source: PeerJ. 2026 Jan 7;14:e20531. doi: 10.7717/peerj.20531 (PMC12790284; doi:10.7717/peerj.20531)
Supplement: Supplemental Information 6 [file peerj-14-20531-s006.docx]

| Table S4 List of annotated genes in *Ficus* chloroplast genome. | | |
| --- | --- | --- |
| Category | Group | Name (s) |
| Photosynthesis | NADPH dehydrogenase | **ndh*A, ***a*ndh*B, *ndh*C, *ndh*D, *ndh*E, *ndh*F, *ndh*G, *ndh*H, *ndh*I, *ndh*J, *ndh*K |
|  | Photosystem I | *psa*A, *psa*B, *psa*C, *psa*I, *psa*J |
|  | Photosystem II | *psb*A, *psb*B, *psb*C, *psb*D, *psb*E, *psb*F, *psb*H, *psb*I, *psb*J, *psb*K, *psb*L, *psb*M*, psb*N, *psb*T, *psb*Z |
|  | ATP synthase | *atp*A, *atp*B, *atp*E, **atp*F, *atp*H, *atp*I |
|  | cytochrome b/f complex | *pet*A, **pet*B, **pet*D, *pet*G, *pet*L, *pet*N |
|  | Rubisco | *rbc*L |
| Self-replication | transfer RNA | ***a*trn*A*-*UGC*,trn*C*-*GCA*,trn*D*-*GUC*,trn*E*-*UUC*,trn*F*-*GAA*,trnf*M*-*CAU*,*  *trn*G*-*GCC*,*trn*G*-*UCC*,trn*H*-*GUG*,*a*trn*I*-*CAU*,**a*trn*I*-*GAU*,*trn*K*-*UUU*,*  a*trn*L*-*CAA*,*trn*L*-*UAA*,trn*L*-*UAG*,trn*M*-*CAU*,*a*trn*N*-*GUU*,trn*P*-*UGG*,*  *trn*Q*-*UUG*,*a*trn*R*-*ACG*,trn*R*-*UCU*,trn*S*-*GCU*,trn*S*-*GGA*,trn*S*-*UGA*,*  *trn*T*-*GGU*,trn*T*-*UGU*,*a*trn*V*-*GAC*,*trn*V*-*UAC*,trn*W-CCA*,trn*Y*-*GUA |
|  | ribosomal RNA | a*rrn*4.5, a*rrn*5, a*rrn*16, a*rrn*23 |
|  | RNA polymerase | *rpo*A, *rpo*B, **rpo*C1, *rpo*C2 |
|  | Small subunit of ribosomal | *rps*2, *rps*3, *rps*4, a*rps*7, *rps*8, *rps*11, ****a*rps*12, *rps*14, *rps*15, **rps*16, *rps*18, *rps*19 |
|  | Large subunit of ribosomal | ***a*rpl*2, *rpl*14, **rpl*16, *rpl*20, *rpl*22, a*rpl*23, *rpl*32, *rpl*33, *rpl*36 |
| Other genes | translational initiation factor | *inf*A |
|  | proteolysis | ***clp*P |
|  | Maturase | *mat*K |
|  | Subunit of acetyl-CoA | *acc*D |
|  | envelop membrane protein | *cem*A |
|  | c-type cytochrome synthesis gene | *ccs*A |
| Genes of unknown function | hypotetical chloroplast reading frames | a *ycf*1, a*ycf*2, ***ycf*3, *ycf*4 |
| * Indicates genes containing one or more introns, a gene with two copies. | | |
